# D. Frank Whitten, D. M. D.

**Published:** 1891-09

**Authors:** 


### Obituary.

### D. FRANK WHITTEN, D.M.D.

Your committee appointed to present resolutions of respect to the memory of the late Daniel Frank Whitten, D.M.D., of Boston, Mass., hereby offer the following:

Whereas, The members of the Massachusetts Dental Society have learned with deep regret of the death of one who was an earnest, capable, and conscientious member of our profession and of this Society. Genial in disposition, whole-souled in every respect, he made a large circle of friends in the various walks of life.

*Resolved*, That in his death the profession has sustained a loss which is most sincerely deplored, that this society has lost a valuable member whose modest demeanor and faithfulness in the discharge of every duty made his character one worthy of our highest esteem, and that the example of his life should be kept before us to urge us to renewed efforts for the good of our profession and the community.

*Resolved*, That this society tenders his family its sincere sympathy in their bereavement.

*Resolved*, That a copy of this preamble and these resolutions be spread upon the records of this Society, that a copy be sent to his family, and to the *Dental Cosmos* and the INTERNATIONAL DENTAL JOURNAL for publication.

(Signed) WALDO E. BOARDMAN,
FREDERIC E. BANFIELD,
HORATIO C. MERIAM,
*Committee.*

